# Piriformis Syndrome and Wallet Neuritis: Are They the Same?

**DOI:** 10.7759/cureus.2606

**Published:** 2018-05-10

**Authors:** Md Abu B Siddiq

**Affiliations:** 1 Physical Medicine and Rheumatology, Brahmanbaria Medical College, Bangladesh

**Keywords:** piriformis syndrome, wallet neuritis

## Abstract

Piriformis syndrome (PS) involves the piriformis muscle and adjacent sciatic nerve, producing features similar to true sciatica of lumbar spine origin, for example, lumbar disc prolapse, thereby confusing pain physicians about the diagnosis. Synonyms that are used frequently for PS are deep gluteal syndrome, extra-spinal sciatica, wallet neuritis, etc. Commonly presenting symptoms of PS include gluteal pain which increases with affected side sitting/per-rectal manoeuvre, and improves with ambulation/intra-lesional steroid, etc. Among various PS associations, wallet neuritis is one of them. However, the latter condition can present with even without the typical features for PS, such as positive flexion adduction internal rotation (FAIR) test, pace sign, etc. In a situation like this, mere discontinuation with fatty buttock wallet is often sufficient for relieving patients’ discomfort, making other approaches unnecessary for the patient (though these approaches are warranted for many PS manifestations). Thus, it would not be wise to use piriformis syndrome and wallet neuritis terminology interchangeably as depicted in many published papers.

## Editorial

Piriformis syndrome (PS) or deep gluteal syndrome is a clinical entity, where presenting symptoms/signs originate from piriformis muscle (PM), or sciatic nerve vicinity, or even both. Patients are usually present with features similar to sciatica, however, a few unique characteristics appear useful in isolating PS from its mimicries; for example, lumbar spinal canal stenosis [1].

PS features aggravate due to prolonged sitting and lying down on the affected side; ambulation, in contrast, improves patients’ sufferings to some extent. Inconsistent clinical manifestations concerning PS make the condition a diagnostic difficulty for the respective physicians’ in most of the cases [1]. The ailment is reportedly common in females, and its prevalence among patients with sciatica diagnosis ranges between 6% and 36% [2]. Nevertheless, it appears as a low back pain association in men of various ages as well, as observed in our recent study [1].

PS is the diagnosis of exclusion of lumbar spine pathology. However, piriformis pathology may be in association with lumbar spine pathology, namely, with lumbar spinal stenosis, as described by Dere et al. in their work. They demonstrated PS and lumbar spinal canal stenosis as examples of double crush syndrome, and situations like this warranted treatment for both conditions, in order to register maximum benefit of the sufferers [1]. Other known associations of piriformis syndrome are myofascial pain syndrome, piriformis pyomyositis, leg-length-discrepancy, PM invasion by carcinoma of the cervix, following deep gluteal intramuscular injection, PM overuse, fibromyalgia syndrome (FMS), etc. [3].

In a published case study, we demonstrated PS in a Bangladeshi woman in association with FMS, as an example of central sensitization of inadequately treated PS. Nevertheless, PS secondary to piriformis pyomyositis, and metastatic cervical lesions are red flag lesions and are associated with raised inflammatory markers. They deserve further meticulous evaluation of the patients to potentiate patients’ maximum recovery and protect them from being treated with inappropriate modalities as well [3].

On the other hand, walletosis, fat-wallet syndrome, or credit-card neuritis have synonymously been used for deep gluteal piriformis syndrome in the previously published write-up. In 1966, through a letter to the editor, a battle was brought to the issue of wallet neuritis in ‘New England Journal of Medicine’; it was mentioned that plastic credit-card stuff made the back wallet abnormally large and caused intense compression on the adjacent sciatic nerve perpetuating manifestations of sciatic neuritis [1]. Further published papers documented heavy wallet loaded with unnecessary scraps (even without credit-card), like papers, visiting cards, etc., that can also proliferate features resembling sciatic neuritis, thereby, replacing the term ‘credit-carditis’ with walletosis. Since then, latter terminology has been used interchangeably with credit-carditis and seems more appropriate [4].

What happens when the fatty wallet compresses ipsilateral sciatic nerve is discussed in our previous publication. We made evidence under NCV study that prolonged wallet-induced pressure caused demyelination of the sciatic nerve in a doctor [1], though without any insult to the adjacent PM as evoked by following maneuvers: FAIR (Flexion Adduction Internal Rotation) test, piriformis sign, Freiberg’s test, Pace sign, and tender per-rectal examination (while the finger glides over the spastic PM), etc., challenging further use of PS and walletosis terminology interchangeably.

PS may be acute or chronic, while in contrast, developing wallet neuritis features is time-consuming, and patients rarely visit the emergency doctors because of this condition. Manoeuvres that are eventful in piriformis muscle pathology are worthless in isolated sciatic nerve pathology; however, pelvis CT (computed tomography) scanning, MRI (magnetic resonance imaging) or MRI neurography, ultra-sonogram, NCS (nerve conduction study) /EMG (electromyogram) of the homo-lateral lower extremity, etc. may be useful in latter condition [1-2]. Nonetheless, piriformis syndrome and walletosis can reportedly be developed in the same patient simultaneously or one can follow another. In our previously published work, we demonstrated a patient presenting with PS symptomology that developed due to his engagement in some household activities, subjecting the lower back and gluteal area to an uncomfortable posture for prolonged period; though, prior to that, the patient had features resembling sciatica that aggravated while sitting on the wallet, especially during driving, restricting him from continuing use of the hip pocket [1]. Likewise, prolonged sitting on the fatty wallet may pose pelvic and gluteal structures including piriformis muscle under exorbitant mechanical strains that induce proliferation of trigger points within the said structure and produce other features for myofascial pain syndrome.

Back pocket heavy wallet-related problems are mounting alarmingly and as per the report, approximately two million Americans are suffering from wallet neuritis. Nevertheless, its epidemiology is not well known in various ethnic groups of different countries. A study regarding the concerned topic is really lacking. Most alarming is that it seems we are yet ready to take the issue seriously [5]. What has been said about the wallet quality in relation to walletosis? To date, the quality of wallet and its association with wallet sciatica have not been studied. We do not know whether the wallet size is a matter of concern causing wallet neuritis features. In 1978, Lutz first demonstrated two cases of credit-card-wallet sciatica that was surfaced on the Journal of American Medical Association and it had been registered that even 28 mm × 37 mm sized wallets were sufficient for proliferating wallet neuritis features [4]. We are yet to know whether any relationship exists between gluteal contour and its how it fits with the wallet. We are in search of answers to the following unanswered quests as well: does wallet texture affect sciatic neuritis perpetuation? Does occupation potentiate developing wallet neuritis features? Or can PS transform into wallet neuritis or vice versa?

Last but not the least, piriformis syndrome is primarily or secondarily a disorder of the piriformis muscle and it may affect sciatic nerve in close proximity; whereas, wallet neuritis is an external compressive neuropathy of the sciatic nerve. Prolonged exposure to the heavy wallet may result in altered alignment of the lumbosacral spinal segment, pelvis, and gluteal anatomical structures including deep-seated piriformis muscle with resultant features characteristic for PS. So, while evaluating walletosis, we must assess whether the adjacent piriformis muscle is involved; and without doing this, it would not be plausible to use ‘piriformis syndrome’ interchangeably with terminology such as ‘walletosis’, ‘credit-carditis’, or ‘wallet neuritis’, etc.
